# A curious case of a spontaneous infective native pseudoaneurysm of the internal carotid artery

**DOI:** 10.1308/rcsann.2023.0012

**Published:** 2023-07-17

**Authors:** ETA Lim, HS Yew, PE Laws

**Affiliations:** ^1^ Christchurch Hospital, New Zealand; ^2^ University of Otago Christchurch, New Zealand

**Keywords:** Carotid, Mycotic, Pseudoaneurysm, Extracranial, Spontaneous

## Abstract

We present a case of a 77-year-old patient who presented with a stroke. She subsequently became unwell and was found to have a spontaneous infective native carotid artery pseudoaneurysm. The patient was managed conservatively as per her wishes. Despite the rarity of this clinical diagnosis, clinicians should be aware of the pathophysiology of this entity and the available literature on management.

## Case history

A 77-year-old woman presented to the emergency department after a collapse and was unresponsive. She was found by her family who witnessed that she was slurring her speech with left upper limb weakness. The patient had a background of a previous left intracranial haemorrhage with residual right-sided weakness, hypertension and a chronic right leg ulcer. Clinical examination demonstrated profound left upper limb weakness but sensation was preserved. Cranial nerve examination was unremarkable. Her white cell count on admission was 7.8 (4–11×10^9^/l) and C-reactive protein was 8 (<5mg/l). Initial computed tomography imaging was normal and the patient subsequently underwent magnetic resonance imaging of the brain which confirmed the presence of an acute infarct.

The patient was admitted under the general medical team and was treated as a stroke. On day 7 after her admission, she became unwell with right ear pain, neck pain and headache. She denied having a sore throat and there was no change to her neurology on examination and no signs of meningism. Otoscopy of the right ear was unremarkable. Examination of her neck demonstrated a firm and tender right neck lump that was hot to touch, but there were no overlying skin changes or erythema. There was no carotid bruit heard on auscultation. There were no peripheral stigmata of infective endocarditis. Cardiac auscultation demonstrated dual heart sounds with a pansystolic murmur. Her known chronic right leg wound was static with no signs of active infection. She was noted to have a fever as high as 39.1°C.

**Figure 1:**
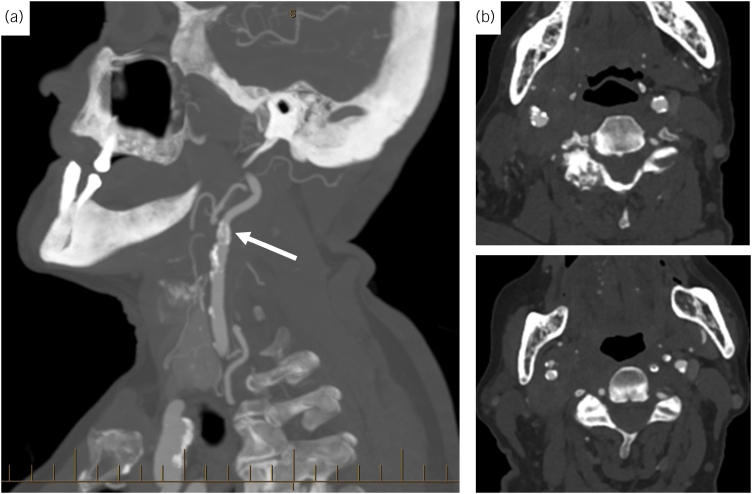
(a) Sagittal view of the computed tomography arteriography (CTA) demonstrating no evidence of an internal carotid artery pseudoaneurysm (white arrow). (b) Axial views of the CTA demonstrating a normal diameter right common carotid and internal carotid artery with no pseudoaneurysm seen.

**Figure 2:**
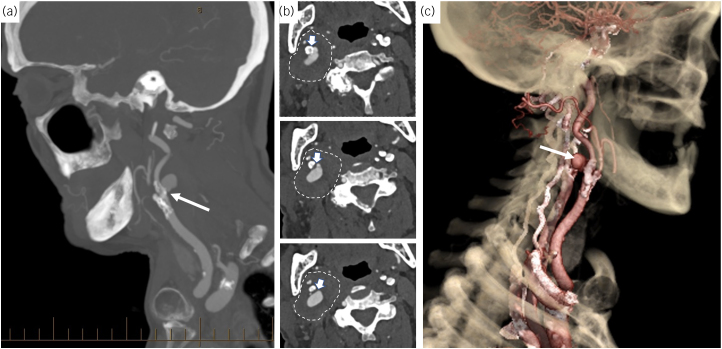
(a) Sagittal view of the computed tomography arteriography (CTA) demonstrating a pseudoaneurysm arising from the proximal portion of the internal carotid artery (white arrow), matched to the same level as Figure 1. (b) Axial views of the CTA demonstrating the formation of a pseudoaneurysm arising posteriorly from the proximal portion of the internal carotid artery (white arrow) with surrounding tissue inflammation (dotted lines), matched to the same level as Figure 1. (c) Three-dimensional reconstruction image demonstrating the pseudoaneurysm arising posteriorly from the right internal carotid artery (white arrow).

Repeat bloods showed an elevated white cell count of 13 (4–11×10^9^/l) and C-reactive protein of 78 (<5mg/l). Blood cultures were done and came back positive for *Staphylococcus aureus*. The patient was commenced on intravenous flucloxacillin. It is likely that the infection originated from the known chronic leg ulcer, which had been swabbed historically and grew *Staphylococcus aureus* as well. A carotid ultrasound was ordered and demonstrated a 13×11mm right internal carotid artery pseudoaneurysm with surrounding inflammation and gas locules, suggestive of an infection. Initial admission imaging was reviewed and interestingly, did not show any abnormality over the carotid artery (Figure 1). A repeat computed tomography arteriography was performed which demonstrated a new 17×8mm right internal carotid artery pseudoaneurysm with adjacent fluid collection containing gas, in keeping with an infective native pseudoaneurysm (Figure 2). Echocardiography showed no evidence of vegetation.

Owing to the patient’s comorbidities and her own wishes to not undergo surgery, she was treated conservatively with intravenous flucloxacillin for 6 weeks duration. A repeat carotid ultrasound was organised 48h following diagnosis of the pseudoaneurysm, which demonstrated a reduction in the size of the infective native pseudoaneurysm. The patient remained well and was transferred to a stroke rehabilitation hospital. She did not progress well with rehabilitation and remained deconditioned. Repeat imaging showed further ischaemic embolic infarcts in the brain. Discussion was held with the patient and family and the patient was palliated. She passed away 47 days later.

## Discussion

Presentation of a spontaneous infective native internal carotid artery pseudoaneurysm is a rare clinical entity. Since the first published case of carotid artery aneurysms in the 1960s, the incidence of this clinical entity is expected to be <1% because presentation of carotid artery aneurysms itself is quoted to be about 1%.^1,2^ Common causes are secondary to trauma or iatrogenic from endovascular interventions or open surgical procedures.^1–3^

Management is complicated by a potential risk of embolisation and rupture, which results in high morbidity and mortality for patients. To our knowledge, there are no available guidelines recommending the best treatment option for these patients. Conservative management alone has been generally discouraged due to the associated complications that can result with an untreated infective native pseudoaneurysm.^4^ Rapid advancement of endovascular technology in the past decade opens up the possibility of performing a carotid covered stent insertion. Open surgery remains the recommended treatment modality by completely removing the infected pseudoaneurysm and performing a primary reconstruction with a vein graft.^1,4,5^

## Conclusion

Spontaneous infective native pseudoaneurysm in the extracranial carotid artery is rare. Prompt treatment in removing the infected tissue is crucial to avoid lethal complications.
